# Monthly Summary

**Published:** 1899-08

**Authors:** 


### Monthly Summary.

EXPERIMENTS WITH NIRVANIN.—In the last publication of the "*Wochenschrift*" my colleague Rotenberger called the attention of the dental fraternity to a new preparation, *Nirvanin.* As I have had some experience with the article, I take the liberty to give my opinion of the same as far as my experiments will warrant. Trials were made partly in collaboration with a practicing physician, Dr. Krueger, and partly alone. We selected the most difficult cases from among the material that came under our care. A number of extractions were made from persons of varying ages after the use of *Nirvanin* with invariably satisfactory results. In two or three per cent. of the cases absolute anesthesia was not attained, but a considerable degree of insensibility resulted from the use of *Nirvanin.* This may have been as much our fault as otherwise, resulting from too quickly applying the forceps. We brushed the gums with a *five per cent. solution* of Nirvanin, or applied tampons saturated, to the gums, which produced the dual effect of anesthesia and antisepsis. According to Professor Einhorn and Dr. Heinz, a one per cent. solution of Nirvanin is sufficient to perfectly prevent the growth of bacteria. The gums were also injected to the periosteum on both sides, the finger gently pressed on the point of injection to prevent an overflow of the liquid and to divide the injected fluid. I discourage the use of old solutions, preferring to make my solution at the time I wish to use it. For convenience sake I have had tablets of Nirvanin made, each containing 0.25 gm., of which I dissolve one or two in 10 c. cm., of water for immediate use. About two or three minutes after this treatment forceps may be applied. No alarming or dangerous symptoms or after-effects appeared or were ever noticed, and no difficulties of any kind resulted except in a case of one patient who had a painless edema during one day. No pain is felt after the operation, and the process of healing is always normal. In all the cases the pulse was good. In several instances we used a ten per cent. solution of Nirvanin to reduce the sensibility of the dentine with most gratifying results. After inserting a tampon saturated with a 10 per cent. solution we could proceed with work in the excavations after a lapse of only two or three minutes. I advise adding 5 per cent. of Nirvanin to temporary fillings, and as a caustic paste that is painless and at the same time anesthetic, I recommend the following:

| | |
|---|---|
| Acid Arsenicos. | 1.0 |
| Nirvanin. | 1.0 |
| Lanoline q. s., to make a paste. | |

I have used Nirvanin in several cases of pulpa-cupping, amputations and root-fillings, but prefer to reserve reports on these cases until later.

Nirvanin, in conclusion, has proven itself in my hands a most effective and lasting anesthetic in the treatment of painful conditions of the mucous membrane of the mouth. After experience with Nirvanin I heartily endorse its use, and wish that others of our profession may make use of it, and report results. Only thus will it be possible to arrive at a correct judgment as to its real efficacy.—Robert Marcus, Dentist, in *Deutsche Zahnaerztichen Wochenschrift No. 39.*

---

TREATMENT OF CARBOLIC ACID POISONING.—Harnsberger (*Charlotte Med. Jour.*, February, 1890), saw a boy, aged 16 years, within thirty minutes of the time that he had swallowed 1.5 ounces of carbolic acid. He was in a limp and comatose state, the pulse being imperceptible. A pint of cream was at once poured into the stomach, which was kneaded in order to mix thoroughly the cream and the carbolic acid. Dry heat and friction were applied to the legs and arms. In two or three hours consciouness returned. The administration of cream and unskimmed milk was continued at short intervals for several hours. The patient entirely recovered in two days. Harnsberger has found that an adult can take four drams of pure carbolic acid mixed with cream and glycerine, or with alcohol, without any toxic symptoms developing.—*Med. News.*

